# Hospital Meetings

**Published:** 1903-09-12

**Authors:** 


### HOSPITAL MEETINGS.

### THE LONDON HOSPITAL.

THE Quarterly Court of the Governors of this hospital was held on Wednesday, the 2nd inst., Mr. J. H. Buxton being in the chair.

The report of the House Committee, after referring to the visit paid by their Majesties on June 11th, stated that the total sum received as the result of the quinquennial appeal was £125,368, towards which the Lord Mayor's appeal had contributed £20,865. The *Sportsman* had consented to open its columns to an appeal on behalf of the hospital, and had issued a circular to this effect to all the cricket and foot-ball clubs in the United Kingdom. Over £100 had already been received in response to this, although it had only been made two or three days. It had been decided to appoint special governors, for this year only, to be called King's Governors, in honour of the Royal visit, who for a donation of £50 would have the privilege of electing a second governor. Good progress had been made with the reconstruction of the hospital, and they trusted soon that the place would be out of the hands of the builders. The donations during the last quarter included one of £10,000 from Mr. E. L. Raphael, £1,000 from Miss Vanderbilt, and £1,000 from Mr. H. Phipps.

The Chairman, in moving the adoption of the report, commented on the very heavy work done by the hospital and said it should be understood that this work, instead of being stationary, was perpetually increasing, and the pressure on the hospital grew daily. Not only did the improved conditions at the hospital induce more patients to apply for its benefits, but it seemed as if East London grew larger and their patients came from greater distances. After referring to the death of Mr. Raphael, the generous donor of £10,000, which was his second donation of a like sum within a few years, the Chairman went on to express the deep sense of gratitude experienced by the governors at the gracious visit paid by the King and Queen. During the last quarter 2,881 in-patients had been discharged, whilst there had been 296 deaths. This was a large number but it proved one thing —that the hospital took in a severer class of cases than any other. The average number of in-patients daily was 687, whilst 22,490 out-patients had been treated as well as 28,000 minor casualties. The cost of reconstruction was very great and would be a heavy responsibility upon them for years to come, especially when so large a sum as £90,000 must be expended yearly on maintenance; he urged the governors to do their utmost to bring the claims of the hospital before their friends.

It was proposed and seconded that Lord Stanley should be elected a member of the House Committee.

